# Hello from the Other Side: How Autoantibodies Circumvent the Blood–Brain Barrier in Autoimmune Encephalitis

**DOI:** 10.3389/fimmu.2017.00442

**Published:** 2017-04-21

**Authors:** Maryann P. Platt, Dritan Agalliu, Tyler Cutforth

**Affiliations:** ^1^Department of Neurology, Columbia University Medical Center, New York, NY, USA; ^2^Department of Pathology and Cell Biology, Columbia University Medical Center, New York, NY, USA; ^3^Department of Pharmacology, Columbia University Medical Center, New York, NY, USA; ^4^Columbia Translational Neuroscience Initiative, Columbia University Medical Center, New York, NY, USA

**Keywords:** blood–brain barrier, autoimmune encephalitis, basal ganglia encephalitis, NMDA receptor, dopamine receptor, autoantibodies, pediatric autoimmune neuropsychiatric disorders associated with streptococcal infections, Sydenham’s chorea

## Abstract

Antibodies against neuronal receptors and synaptic proteins are associated with autoimmune encephalitides (AE) that produce movement and psychiatric disorders. In order to exert their pathological effects on neural circuits, autoantibodies against central nervous system (CNS) targets must gain access to the brain and spinal cord by crossing the blood–brain barrier (BBB), a tightly regulated gateway formed by endothelial cells lining CNS blood vessels. To date, the pathogenic mechanisms that underlie autoantibody-triggered encephalitic syndromes are poorly understood, and how autoantibodies breach the barrier remains obscure for almost all AE syndromes. The relative importance of cellular versus humoral immune mechanisms for disease pathogenesis also remains largely unexplored. Here, we review the proposed triggers for various autoimmune encephalopathies and their animal models, as well as basic structural features of the BBB and how they differ among various CNS regions, a feature that likely underlies some regional aspects of autoimmune encephalitis pathogenesis. We then discuss the routes that antibodies and immune cells employ to enter the CNS and their implications for AE. Finally, we explore future therapeutic strategies that may either preserve or restore barrier function and thereby limit immune cell and autoantibody infiltration into the CNS. Recent mechanistic insights into CNS autoantibody entry indicate promising future directions for therapeutic intervention beyond current, short-lived therapies that eliminate circulating autoantibodies.

## Introduction

Antibody-mediated central nervous system (CNS) autoimmunity, the hallmark of several autoimmune encephalitis (AE) syndromes that produce movement and psychiatric disorders, is initiated when antibodies recognize neuronal receptors or synaptic proteins as foreign proteins (1–3). Antibodies directed against neuronal antigens can arise in the periphery when target proteins are expressed ectopically on tumor cells. AE has also been linked to various infectious triggers, including bacteria, viruses, fungi, and other parasites. However, an underlying infection is the causative agent for only a small fraction of all AE cases; in many cases, the triggers are either unknown or unidentified infectious agents (4, 5). While the evidence supporting various microbial triggers is scant, some autoimmune encephalopathies have well-established infectious links. For example, untreated Group A *Streptococcus* (*S. pyogenes*) infections cause autoimmune sequela in many target tissues such as the heart or CNS, manifested as either rheumatic fever or Sydenham’s chorea (SC), respectively (6–8). Several recent reviews have highlighted the clinical and mechanistic features for many of these currently accepted autoimmune encephalitides and the aberrant autoimmunity/CNS axis (4, 5, 9–15). Here, we review infectious and non-infectious triggers for AE and discuss findings from animal models of AE related to blood–brain barrier (BBB) integrity, immune cell infiltration into the CNS, and neuronal circuit dysfunction that provide useful avenues to improve diagnosis (e.g., clinical assays and imaging techniques). We will then outline routes for antibody and immune cell entry into the CNS, with a focus on the predominant pathways leading to BBB breakdown that allows entry of autoantibodies into the CNS. Finally, we will briefly review current therapies for selected AEs and propose future treatment options aimed at preventing autoantibody entry. Recent advances point to an underlying autoimmune etiology that may be relevant for many movement and neuropsychiatric diseases (2, 4, 16). Thus, elucidating the mechanisms for autoantibody access to the CNS may provide a wider spectrum of treatment options for patients with these complex and puzzling disorders.

## Autoimmune Encephalitis Triggers

Autoimmunity against brain targets is inherently mysterious, because traditional thinking holds that the immune system minimally surveys the CNS compared to other organs. Non-CNS self-antigens are selected against to maintain tolerance; however, CNS antigens have been thought to be excluded from immune monitoring (17). Although this thinking has been recently challenged with the identification of both glymphatic and lymphatic circulation in the CNS (18, 19), re-exposure of the immune system to brain antigens, or the presence of an outside trigger that causes production of cross-reactive autoantibodies, is a crucial step in CNS autoimmune disease.

### Bacteria

Perhaps the earliest identified CNS autoimmune disease is linked to an infectious trigger: untreated Group A *Streptococcus* (GAS or *S. pyogenes*) infections can in some cases give rise to SC, in which antibodies directed against a streptococcal surface protein cross-react with brain antigens (20). While SC is characterized by movement difficulties (chorea) affecting both gait and the tone of large muscle groups, more recently, a group of children within an SC cohort displaying both prominent psychiatric symptoms and fine choreiform movements prompted the recognition of a new syndrome: pediatric autoimmune neuropsychiatric disorders associated with streptococcal infections (PANDAS) (21). Both SC and PANDAS are part of a group of basal ganglia autoimmune encephalopathies (BGE) for which the humoral adaptive immune response (i.e., autoantibodies) plays an important role in disease pathogenesis, as described in human patients as well as rodent models (3, 16). In most cases, a recent GAS exposure or infection can be identified in afflicted children and subsequent exposures typically prompt an acute exacerbation of symptoms (22). Anti-GAS titers correlate with symptom severity in many but not all SC and PANDAS cases (23). Anti-neuronal autoantibodies that erroneously recognize dopamine D1R/D2R receptors or other neuronal targets in the basal ganglia have been identified in sera from patients with SC or PANDAS, and they respond positively to immune therapies such as intravenous immunoglobulin (IVIg) or plasmapheresis, consistent with an autoimmune mechanism (7, 16, 21, 24–30). The autoimmune mimicry hypothesis, namely that antibodies generated from an aberrant humoral immune response to *S. pyogenes* infections recognize host-specific proteins due to epitope similarity, has been proposed to underlie the secondary sequela in BGE (20, 31). However, this hypothesis assumes that BBB permeability is impaired to allow antibody entry into the CNS, because BGE occurs in the absence of brain infection.

Infections by *Campylobacter jejuni* and, in rare cases, by other bacteria (32, 33), induce Guillain–Barré syndrome (GBS) and the atypical Guillain–Barré-related diseases [Miller Fisher syndrome (MFS) and Bickerstaff brainstem encephalitis (BBE)], whose symptoms lie on a continuum with traditional GBS including prickling, weaknesses in extremities, motor deficits, and pain (34–36). While these diseases are caused by the same autoantibodies against gangliosides (GD3, GQ1b, GM1, or GT1a), GBS and MFS affect peripheral nerves whereas BBE affects primarily the CNS (37, 38). Blood vessels in peripheral nerves are protected by a blood–nerve barrier (BNB) that has some similarities to the BBB (39–41). Although the BNB can be disrupted by autoantibodies present in sera from patients with multifocal motor neuropathy (42), this review is primarily focused on autoantibody entry into the CNS across the BBB rather than PNS across the BNB.

### Viruses

Viruses have been proposed to initiate some autoimmune encephalopathies. In systemic lupus erythematosus (SLE), autoantibodies cross-reacting with Epstein–Barr nuclear antigen-1 and the 60 kDa Ro protein target a variety of organs, including the CNS (31). Anti-Ro antibodies are frequently generated and detected early in clinical SLE, making them attractive candidates for an initiating autoantigen. Other viruses implicated in neuropsychiatric disease include influenza, herpes virus-1 and -2, Epstein–Barr virus, and bornavirus (43, 44). Herpes simplex encephalitis has been linked to subsequent development of NMDAR encephalitis in some cases (2, 9). Notably, the majority of viral triggers are hypothesized to create a pro-inflammatory state that “primes” the immune system, including CNS-resident immune cells termed microglia, to become overactive leading to an autoimmune response against the CNS (2, 45). This contrasts with the molecular mimicry hypothesis proposed for *S. pyogenes*-induced BGE, in which antibodies directed against bacterial surface antigens cross-react directly with self-antigens (20, 31).

### Tumors

In contrast to molecular mimicry, production of antibodies against a brain antigen can occur in the periphery when a tumor cell expresses surface proteins found in the brain. Tumor masses are known to express a wide variety of non-tissue-specific surface proteins, including neuronal antigens. The original cohort of NMDA receptor encephalitis (NMDARE) patients contained young women bearing ovarian teratomas that express NMDAR, thus initiating peripheral immune activation (46). Subsequent BBB permeability by an unknown mechanism would then allow NMDAR antibodies to target glutamatergic synapses also containing this receptor. However, tumors are not present in all cases of NMDARE, and indeed, the tumor rate is approximately 50% for such patients (47). Anti-GAD65 antibodies, which are the putative culprits underlying cerebellar ataxia, stiff person syndrome, and Batten’s disease (3), are also associated with neoplasms that aberrantly express GAD65, albeit at lower frequency than those producing NMDAR antibodies (48, 49). Finally, serum antibodies targeting the voltage-gated potassium channel (VGKC) complex may be also linked to tumors. These include antibodies against leucine-rich glioma-inactivated 1 (LGI1), contactin-associated protein 2 (Caspr2), and contactin 2 as well as those recognizing the entire VGKC protein complex. IgGs targeting the VGKC complex have been found in sera (50, 51) and cerebrospinal fluid (CSF) (51) from patients with both limbic encephalitis and Morvan syndrome (VGKC complex encephalitis, peripheral nerve hyperexcitability) (52), and Morvan syndrome has been linked to thymoma in 37% of patients (53). Conversely, anti-VGKC complex antibodies are present in sera from 32% of patients with verified thymoma, the majority of whom develop myasthenia gravis, which targets acetylcholine receptors on muscles that are supplied by blood vessels lacking a tight endothelial barrier (54). Since only a minority of patients with VGKC complex antibodies have co-occurring thymoma, this suggests that other mechanisms underlie development of these autoantibodies, similar to NMDARE. In addition, the clinical features accompanying patients with VGKC antibodies are highly variable (52). VGKC antibodies may, therefore, be useful biomarkers for inflammatory neurological disease in general and are potentially associated with the presence of other autoantibodies or immune cell infiltration into the CNS (52).

## Animal Models for CNS Autoimmune Disorders

Numerous rodent studies have expanded our understanding of the molecular events involved in antibody generation and pathogenicity for several varieties of AE, shedding light on the mechanisms of immune cell infiltration and barrier breakdown. Rodent models for NMDARE, BGE, GAD65 encephalitis, BBE, and Morvan syndrome vary widely in both their methods of antigen exposure and the disease parameters recapitulated (see Table 1 for summary). A majority of the studies surveyed here use animal models in which disease is initiated either by exposure to an initial trigger (bacteria or virus) in the periphery or by direct infusion into the CNS of sera or purified autoantibodies isolated from patients, thereby circumventing the BBB.

**Table 1:** **Summary of published rodent models for several autoimmune encephalitides**.

Disease modeled	Strain/species (sex)	Autoantibody source	Delivery	Immune response	Neural consequences	Reference
Sydenham’s chorea/pediatric autoimmune neuropsychiatric disorders associated with streptococcal infections	SJL mouse (F)	Unknown	Intranasal infection with *S. pyogenes*	Microglial activation and infiltrating CD4^+^ T cells in olfactory bulb (OB)	Decreased excitatory synapse proteins in OB glomeruli; blood–brain barrier breakdown in OB, lateral hypothalamus, and amygdala	(55)
	Lewis rat	Induced in model	Subcutaneous immunization with *S. pyogenes* emulsion	Autoantibodies detected against tubulin	Dopamine D2-dependent compulsive grooming; impaired motor coordination; IgG deposition in striatum, thalamus, and cortex; IgG-induced elevation of CaMKII signaling in cultured neurons	(56)
	SJL mouse	Induced in model	Subcutaneous immunization with *S. pyogenes* emulsion	Specific increase in IgG1 subclass; no change in IgG2 nor IgG3 pool	Increased rearing; decreased motor coordination; impaired olfactory discrimination; improved spatial memory performance; IgG deposition in striatum and cerebellum	(57)
	SJL mouse (M)	Adoptive transfer from immunized cohort	Intravenous injection paired with intraperitoneal lipopolysaccharide injection	Not analyzed	Increased rearing; IgG deposition in dentate gyrus	(57)
	Lewis rat (M)	Induced in model	Subcutaneous immunization with *S. pyogenes* emulsion	Autoantibodies against D1R, D2R, and serotonin receptors	Impaired motor coordination; compulsive grooming	(58)
	Lewis rat (M)	Adoptive transfer from immunized cohort	Intra-striatal infusion	Not analyzed	Impaired motor coordination; IgG deposition in striatum	(58)
	SJL mouse (M)	Induced in model	Subcutaneous immunization with *S. pyogenes* emulsion	Microglial activation in white matter tracts; infiltrating CD3^+^ T cells	Impaired motor coordination; repetitive behaviors; increased rearing; excessive lactate; blunted startle response (PPI)	(59)

NMDA receptor encephalitis	Lewis rat	Patient cerebrospinal fluid (CSF)	Bath application to cultured neurons	Not analyzed	Autoantibody-mediated internalization of NMDAR from synapses; selective loss of NMDA-mediated currents	(60)
	Lewis rat (F)	Patient CSF	Intrahippocampal infusion	Not analyzed	Decreased NMDAR density in hippocampus	(60)
	C57BL/6 mouse	Patient sera	Intraventricular injection	Not analyzed	IgG deposition in hippocampus; more seizures and higher seizure scores after pro-convulsant challenge; no change in total NMDAR number	(61)
	C57BL/6N (ApoE^−/−^) mouse (M)	Patient sera	Intravenous injection	All autoantibody isotypes affect behavioral assessments and endocytosis	Decreased spontaneous locomotion and increased MK-801-evoked locomotion in ApoE^−/−^ mice, but not WT, treated with autoantibody; increased endocytosis by cultured neurons after autoantibody treatment	(62)
	C57BL/6J mouse	Patient CSF	Intraventricular infusion	Not analyzed	Reversible memory deficits, anhedonia, and depressive-like behavior without locomotor impairment; hippocampal IgG deposition; decreased NMDAR densityin hippocampus	(63)

Stiff person syndrome/cerebellar ataxia	Wistar rat (M)	Patient sera	Intracerebellar infusion	Not analyzed	Decreased potentiation from excitatory stimulus trains; decreased NMDA-mediated NO synthesis	(64)
	Wistar rat (M)	Patient sera	Lumbar paraspinal injection	Not analyzed	Abnormal high baseline activity; increased excitability of anterior horn neurons	(64)
	Lewis rat (F)	Patient sera	Intrathecal infusion	Not analyzed	Recapitulation of paralysis; autoantibody-mediated internalization of amphiphysin on GABAergic neurons; decreased GABA release from cultured neurons; increased IPSC frequency and amplitude recorded *in vivo* from hippocampal granule cells	(65)
	Lewis rat (F)	Patient CSF	Intrahippocampal injection	Not analyzed	No changes in evoked and spontaneous GABAergic transmission in CA1 neurons	(66)
	Cultured mouse hippocampal neurons	Patient sera	Bath application	Not analyzed	No changes in evoked and spontaneous GABAergic transmission in cultured hippocampal networks	(67)

### *S. pyogenes*-Triggered BGE

**Figure 1:**
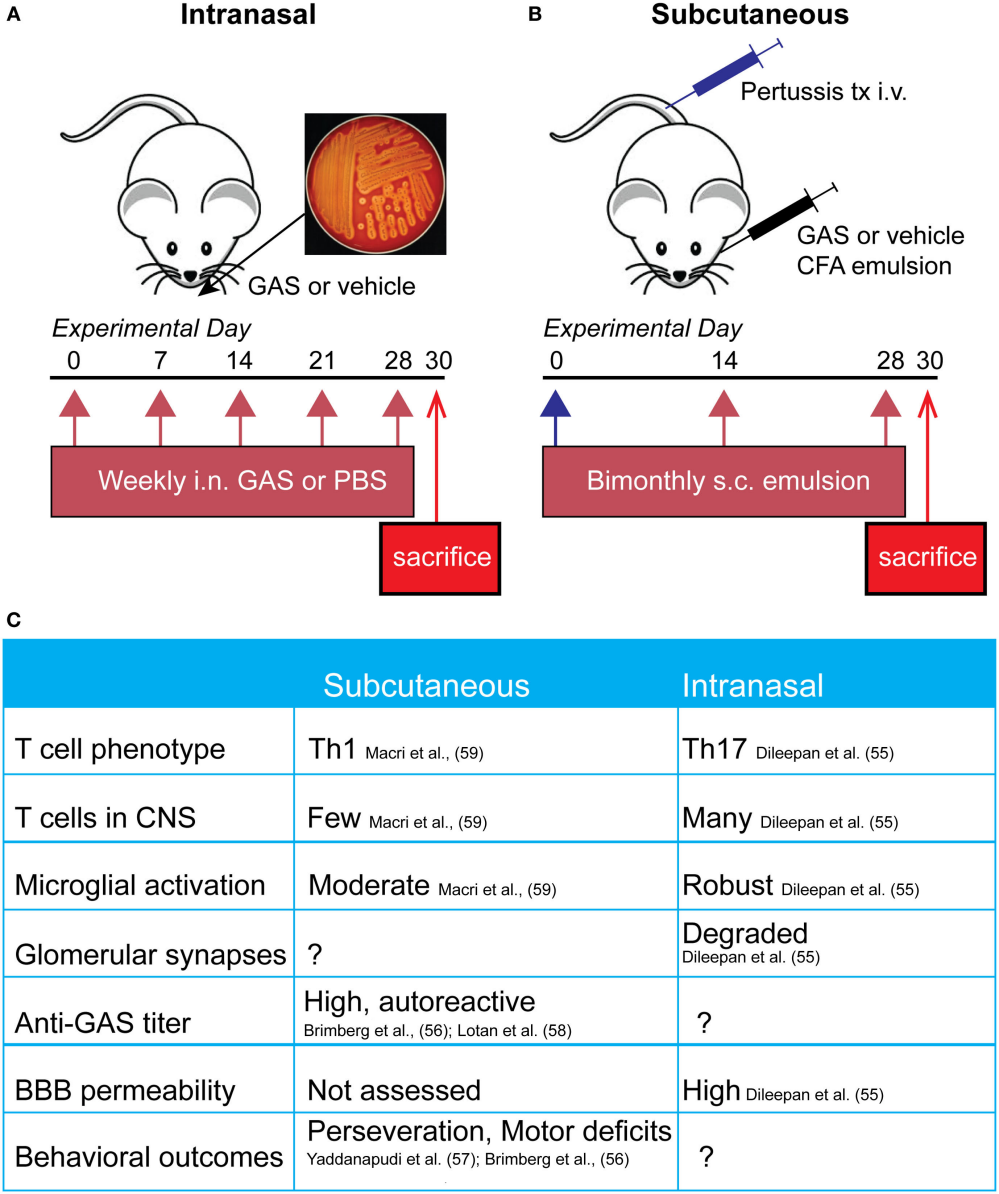
**Comparison of mouse pediatric autoimmune neuropsychiatric disorders associated with streptococcal infections (PANDAS)/Sydenham’s chorea (SC) models**. **(A)** Schematic representing the initiation of the intranasal model, where mice receive live bacteria intranasally once a week for 5 weeks prior to sacrifice. **(B)** Subcutaneous GAS exposure involves adjuvant and antigen exposure three times, every 2 weeks, following an initial boost with intravenous pertussis toxin. **(C)** Comparison of immune, neural, and behavioral outcomes after each route of GAS exposure. Investigators have used either subcutaneous or intranasal routes to induce an immune response against *S. pyogenes* [Group A *Streptococcus* (GAS)] in efforts to understand the mechanisms underlying the behavioral and motor symptoms characteristic of PANDAS and SC patients. The former route necessitates opening the blood–brain barrier (BBB) artificially using *B. pertussis* toxin, whereas the latter features intranasal inhalation of live bacteria to trigger a Th17 response in nasal tissue that is directly communicated to the brain along the olfactory nerve.

**Figure 2:**
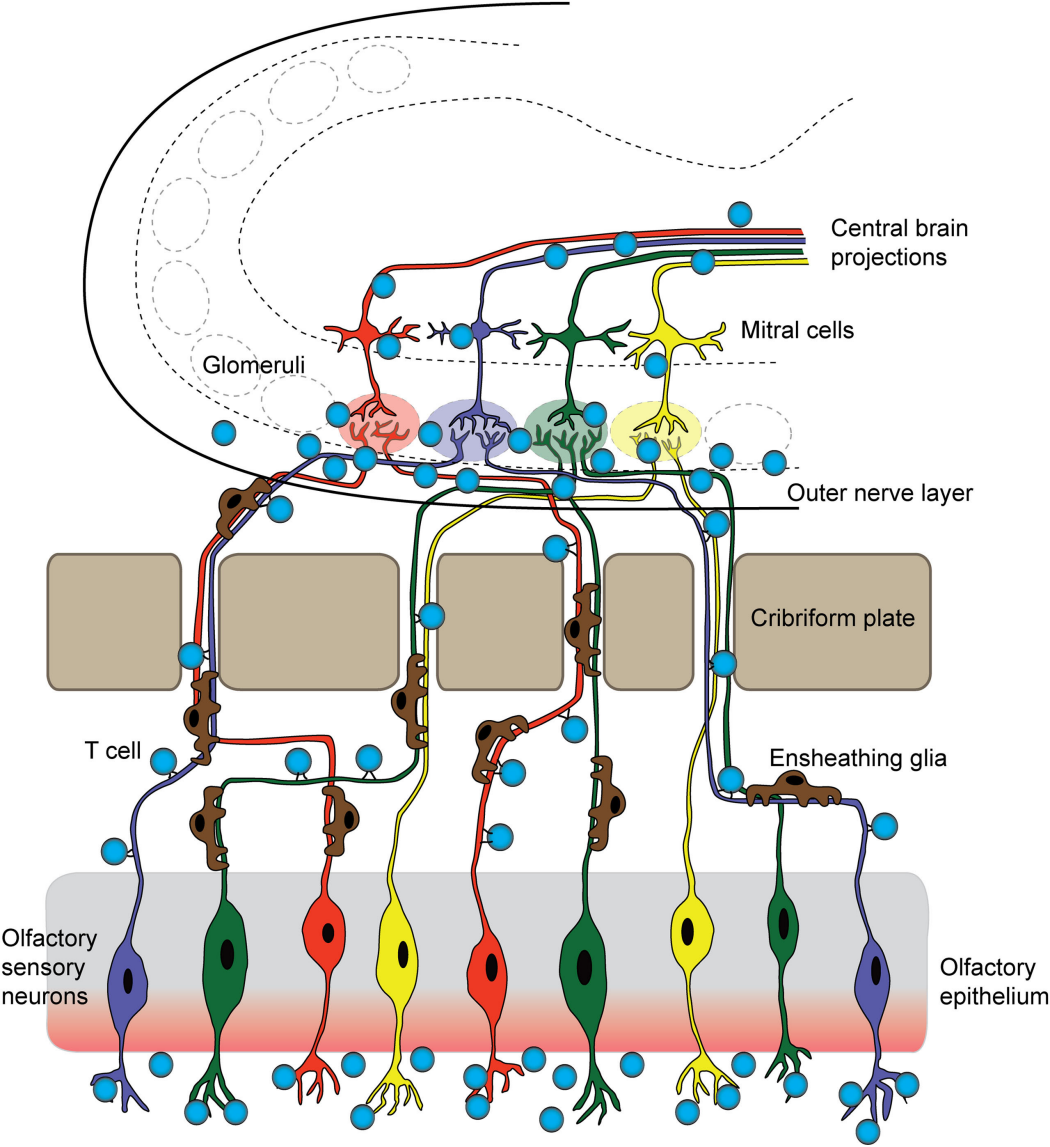
**T cells originating in the nose infiltrate the brain parenchyma**. In a mouse model for pediatric autoimmune neuropsychiatric disorders associated with streptococcal infections, T cells first arise in the nasal-associated lymphoid tissue and olfactory epithelium at the site of a latent *S. pyogenes* infection. These cells then respond to chemotactic cues release by olfactory ensheathing glia to accompany sensory axons into the brain. Once there, infiltrating T cells release inflammatory cytokines and chemokines, damaging synapses within olfactory glomeruli and breaking down tight junctions of olfactory bulb capillaries. These T cells may then move centrally, against the rostral migratory stream and toward the SVZ, and exit through the ventricles, or continue following the projections of olfactory mitral/tufted neurons.

Animal models for post-streptococcal BGE have been focused on demonstrating the ability of GAS to prime development of an autoimmune reaction by stimulating adaptive cellular and humoral immune responses. In the mouse, intranasal (i.n.) infections with live bacteria polarize T cells located in the nasal-associated lymphoid tissue (NALT, the mouse structural analog of human tonsils and adenoids) toward a Th17 phenotype, a T cell subtype that is both essential for mucosal immune protection against bacteria but also strongly implicated in many autoimmune diseases (Figure 1A). Multiple i.n. *S. pyogenes* infections strengthen this Th17 immune response, largely due to induction of IL-6 and TGF-β1, which are two pro-inflammatory cytokines essential for Th17 differentiation (68). IL-6 is essential for clearance of bacteria after i.n. infection, *via* generation of Th17 cells; *IL-6^−/−^* mice are capable of generating a Th1 immune response to an i.n. bacterial challenge but cannot control infection (69). This model has been used to demonstrate that repeated i.n. infections with *S. pyogenes* induce migration of GAS-specific Th17 cells and other T cell subtypes from the nasal epithelium to the olfactory bulb (OB) (Figure 2), where sensory axons make connections with projection interneurons to form the neural circuitry essential for odor discrimination, as well as to other CNS regions (55). The presence of *Streptococcus*-specific Th17 cells in the CNS after repeated i.n. infections increases the permeability of capillaries in several CNS regions, including the OB, amygdala, and hypothalamus, thereby enabling deposition of serum IgGs and potential anti-CNS autoantibodies. This is largely due to disruption in the organization of tight junction (TJ)-associated proteins, which control an essential aspect of BBB function (55) (see below for a more detailed discussion). The intranasal model produces profound changes in olfactory neural circuitry by reducing vGluT2 expression and thus excitatory input at the presynaptic terminals of olfactory sensory axons and perturbing the excitatory/inhibitory balance within the primary olfactory circuit (55). This model of post-*S. pyogenes* autoimmunity demonstrates a central role for the cellular adaptive immune response (e.g., bacterial-specific Th17 cells in the CNS) in disrupting BBB function, thus promoting entry of antibodies into the CNS and inducing changes in synaptic signaling. Although such a cellular adaptive immune response has not been identified to date in the nervous systems of children suffering from BGE, *S. pyogenes*-specific Th17 cells can be found in the tonsils of human patients (55), making Th17 lymphocytes a potential causative agent in either initiation or persistence of BGE disease pathogenesis.

A second group of rodent models for BGE employs subcutaneous immunization with an antigenic target (bacterial homogenate) plus complete Freund’s adjuvant to activate the immune system, in conjunction with agents (i.e., *B. pertussis* toxin) that open the BBB to provide access to brain targets (Figure 1B). In this model, mice and rats develop a strong humoral immune response toward *S. pyogenes* and show behavioral abnormalities. Specifically, GAS-immunized rodents display increased rearing and decreased locomotion, as well as increased repetitive and perseverative behaviors, impaired pre-pulse inhibition, and reduced concentrations of serotonin in the prefrontal cortex as compared to controls (56, 57, 59, 70). Moreover, adoptive transfer of serum IgGs from *S. pyogenes*-immunized mice to naive recipient mice, or direct infusion of sera into rat brains, recapitulates some of the behavioral deficits in recipient rodents, whereas no effects were observed after adoptive transfer of IgG-depleted serum (57, 58). Histological examination of brain tissue revealed antibody deposition in the deep cerebellar nuclei and hippocampus in mice and the striatum, cortex, and thalamus in rats (56, 57, 70). Serum IgG isolated from immunized rodents recognizes both cerebellar targets and human D1/D2 dopamine receptors by either western blotting or ELISA (56, 57). There is a high variability among the mouse and rat BGE models. This may reflect differences in key immune-related genes between species, especially those related to T cell function or within the MHC locus for antigen presentation and immune cell stimulation. All studies used inbred animals (Lewis rats and SJL or C57BL/6 mice); therefore, intra-strain variability is low in these highly variable regions; however, differences between strains and species may be more pronounced. It is also possible that differences observed in mouse and rat studies are due to variability in their humoral immune response to GAS or differences in immunization protocols. Taken together, subcutaneous animal models for BGE have provided useful information regarding the humoral immune response after bacterial infection (i.e., the presence of antibodies directed against GAS and CNS) and demonstrate a clear link between *S. pyogenes* exposure and behavioral abnormalities (see Figure 1C for comparison of intranasal and subcutaneous models). However, these are somewhat artificial models for immune system activation, because human GAS infections occur primarily by the i.n. route. Moreover, subcutaneous animal models for BGE leave unanswered an important question for disease pathogenesis, namely how these autoantibodies can penetrate the CNS since the BBB is artificially opened (71).

### NMDA Receptor Encephalitis

Most animal models for NMDARE involve infusing serum antibodies from acutely ill patients into the rodent brain in an attempt to replicate behavioral symptoms of the human disease. Infusion of autoantibodies from NMDARE patients into the lateral ventricles of mice causes impaired recognition memory after 10 days of infusion, which is reversible after antibody washout (63). Histologically, patient IgG binding is strongest in the hippocampus, which has a very high concentration of NMDARs, supporting the conclusion that internalization of NMDAR in this region may underlie memory deficits with minimal effects on aggression, locomotion, and anxiety-like behavior (61, 63). Intraventricular delivery of NMDAR antibodies also decreases seizure thresholds after administration of the convulsant pentylenetetrazol, resulting in more frequent and stronger seizures in NMDAR antibody-recipient mice (61). These data mirror the course of human disease, because NMDARE patients frequently present with seizures in conjunction with memory loss, hallucinations, and anxiety (72). Using the electroencephalograph (EEG), NMDARE patients show unusual delta rhythmic activity with superimposed beta or gamma activity (72). Similar spike events are also detectable using EEG during seizures in mice after NMDAR antibody infusion. Electrophysiological changes are also apparent in hippocampal neurons after intrahippocampal infusion of anti-NMDAR antibodies (73). Patient-derived NMDAR antibodies decrease NMDAR-dependent hippocampal long-term potentiation (LTP) in a manner similar to antibodies directed against extracellular domains of the NR2 or NR1 subunits of the NMDAR. After anti-NMDAR antibody treatment, dentate granule cells show smaller evoked responses to stimulation, increased spike thresholds after EPSP, impaired LTP, and decreased NMDAR densities in postsynaptic areas (60). In summary, there is strong evidence that antibody binding to the NMDAR leads to cross-linking and internalization but not necessarily destruction of the receptor, causing deficits in synaptic transmission that include LTP. NMDAR antibody deposition is strongest in the hippocampus after intraventricular infusion, indicating that deficits in perforant pathway may be the most prominent in this animal model for NMDARE. However, this model bypasses the BBB. In an elegant series of experiments, Hammer and colleagues showed that the BBB efficiently excluded NMDAR antibodies from the hippocampus if delivered intravenously (i.v.) in healthy mice. However, in *ApoE^−/−^* mice that have a defective BBB, intravenously injected anti-NMDAR antibodies can access brain antigens, and mutant mice injected i.v. with anti-NMDAR antibodies from patient sera show decreased locomotion as compared to those treated with control sera (62). Therefore, there is a clear need to develop animal models for NMDARE that incorporate mechanisms that disrupt BBB integrity to more closely mirror the human syndrome.

### Anti-GAD65 Antibodies

Autoantibodies against proteins involved in GABAergic transmission, including anti-glutamic acid decarboxylase (GAD) 65, anti-GAD67, and anti-amphiphysin, are the putative autoantibodies for cerebellar ataxia, stiff person syndrome, and Batten’s disease (3, 74). Animal models using intraventricular infusion of anti-GAD65 antibodies isolated from patients have provided conflicting results. Bath application of such antibodies causes increased IPSP frequencies in cultured hippocampal neurons (75). Cerebellar infusion of anti-GAD65 antibodies in rodents also leads to reduced GABA synthesis in cerebellar basket cell terminals, resulting in disinhibition of Purkinje cells. However, hippocampal infusion of anti-GAD65 antibodies *in vivo* yields no changes in synaptic transmission assessed with physiological measurements in hippocampal slice preparations, whereas anti-NMDAR antibodies are sufficient to induce defective LTP (66). It is possible that anti-GAD65 antibodies do not gain access to their intracellular antigen after infusion in the hippocampus, or that pathogenic antibodies are in fact directed against a different antigen. Antibodies against amphiphysin rather than GAD65 have been shown to induce stiff-person syndrome-like symptoms in rats (65). Methodological differences and debate about pathogenicity of GAD65 antibodies has complicated the interpretation of these studies, because GAD65 antibodies are also prevalent, albeit at lower concentrations, in type 1 diabetic patients who have no neurological abnormalities. Careful screening of patient antibody samples is, therefore, necessary for anti-GAD65 collections, in order to exclude the likely non-pathogenic antibodies that are present in patients with diabetes.

### Anti-GQ1b Antibodies

There are few studies on the CNS effects of anti-GQ1b antibodies, the pathogenic basis for BBE, which hinders our understanding of this disease; however, there have been some reports on their binding location and disease mechanism. Anti-GQ1b antibodies were shown to induce complement deposition; the combination of antibody and complement is necessary to disrupt neuromuscular junction function *in vivo* (76). Binding of the antibody-complement complex induces local Ca^2+^ flux into neurons *in vitro*, which in turn alters mitochondrial function and causes hydrogen peroxide production in addition to neuronal excitation (76). Mechanistically, this provides an interesting perspective on GBS and related syndromes, which lie at the intersection of innate and adaptive immune mechanisms. However, other than lesions identified by MRI in human MFS and BBE patients, there has been no investigation into how anti-GQ1b antibodies enter the CNS to affect brain function (77, 78).

### Anti-VGKC Complex Antibodies

The VGKC complex contains a signaling protein tetramer as well as several scaffolding proteins. Early identification of antigenic targets had attributed antigenicity to the potassium channel K_v_1.1/1.2 itself, while more recent work argues that the antibody targets are more likely the associated scaffold proteins (LGI1, Caspr2, and contactin-2) that precipitated along with the channel during the original identification (79). Most investigation into VGKC antibodies has used rodent tissue or cultured cells to test for reactivity of patient antibodies that are harvested from CSF or serum. Sera from both AE and neuromyotonia patients react with rat (80) and mouse (81) hippocampal sections, as well as with the potassium channels K_v_1.1 and K_v_1.2 transfected into HeLa cells (80), but not with *Caspr2^−/−^* mouse hippocampal sections (81). One rodent study used adoptive transfer of patient plasma or purified IgG into mice to address how these antibodies alter neuronal transmission in peripheral nerves (82). After repeated injections with patient IgG, mice showed no clinical symptoms but had increased quantal size and potassium-dependent increases in EPP amplitude in peripheral nerves as compared to control mice (82). While there has been much progress in identifying the target antigen of these antibodies, many questions about their pathogenicity and CNS access remain unanswered.

## Potential Routes of Antibody Entry into the CNS

### Blood–Cerebrospinal Fluid Barrier (BCSFB)

**Figure 3:**
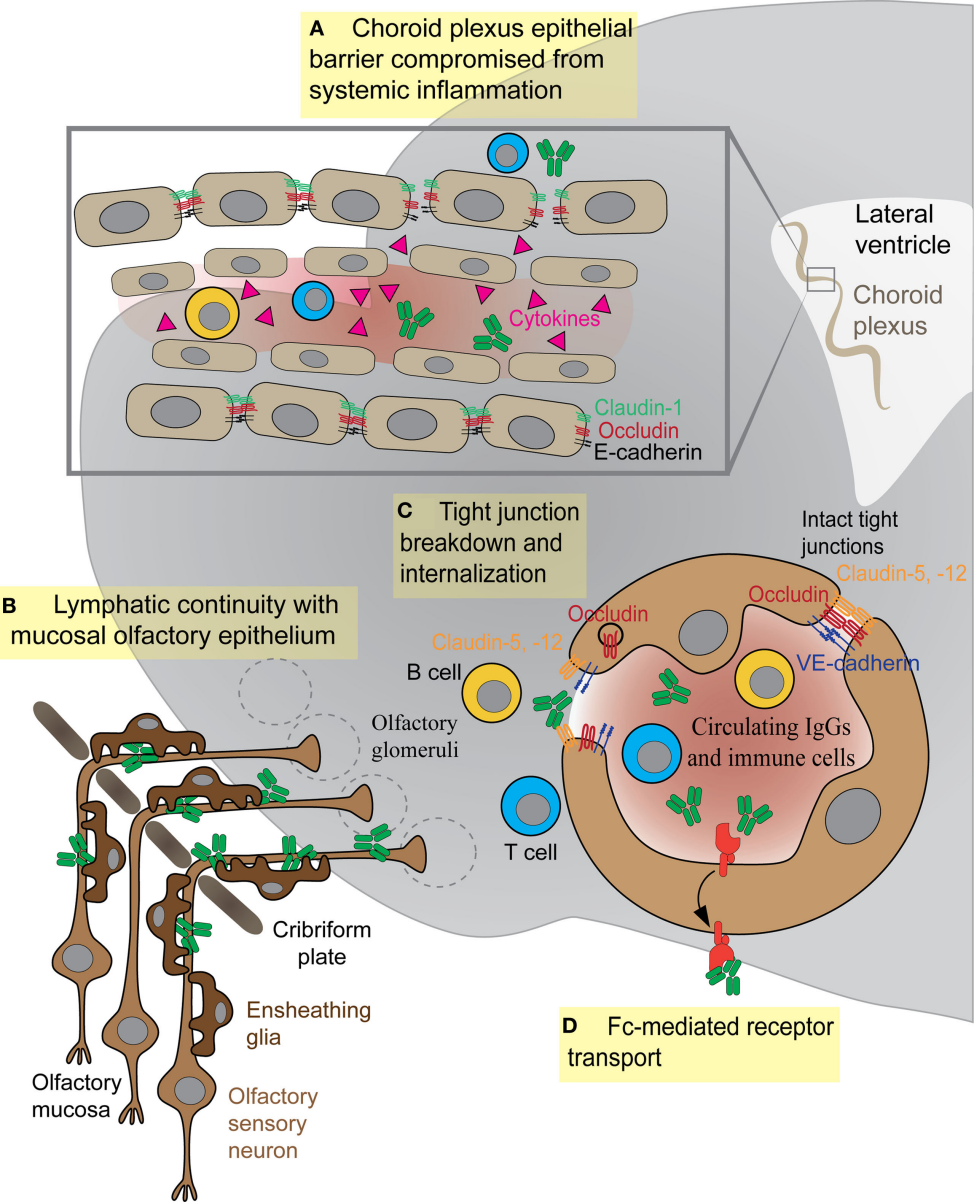
**Antibody and immune cell access to the brain parenchyma *via* four distinct routes**. **(A)** Systemic cytokines break down tight junctions (TJs) within the brain–cerebrospinal fluid barrier to allow central nervous system (CNS) access of antibodies or immune cells. **(B)** Olfactory ensheathing glia facilitate transport of IgGs or immune cells along sensory axons exiting the olfactory mucosa. **(C)** Inflammatory cytokines in the bloodstream damage TJs between endothelial cells, thus allowing antibodies or immune cells (T or B cells) to enter the CNS. **(D)** F_c_ receptor directionality reverses, shuttling IgG from vessels into brain parenchyma as in systemic lupus erythematosus.

Epithelial cells within the choroid plexus form a tight barrier termed the BCSFB that restricts diffusion of serum proteins and immune cells from the more leaky blood vessels of this tissue into the CSF (83). The molecular composition of TJs within the BSCFB is less well understood than those comprising the BBB. However, several key junctional proteins that regulate paracellular permeability across epithelial cells of the choroid plexus are present at high levels in both embryonic and adult stages, suggesting that this barrier matures early during development (84). The critical function of these junctions is to create a physical barrier to paracellular diffusion, allowing cells to become polarized with distinct luminal and abluminal components. In addition, epithelial cells of the BCSFB also express specialized transporter proteins to allow transit of certain plasma proteins across this barrier (84, 85). The BCSFB is less tightly regulated than its brain counterpart and can become more permeable to immune cells or antibodies during disease states. Th17 lymphocytes cross the BCSFB several days prior to BBB damage, in order to enter the CNS and initiate their immune attack during experimental autoimmune encephalitis (EAE), a mouse model for human multiple sclerosis (86). This entry appears to be an essential step in initiating the CNS immune response, because CCR6 receptor-deficient Th17 cells that cannot cross the BCSFB do not induce EAE (86). In mouse models for SLE, the cell adhesion molecules vascular cell adhesion molecule-1 (VCAM-1) and intercellular adhesion molecule 1 (ICAM-1) show elevated expression in the choroid epithelium and promote large amounts of cellular infiltrates (T and B cells) in the choroid (87, 88). The role of such infiltrating immune cells is still debated, but the BCSFB epithelium is clearly activated and disrupted by increased cytokine expression (87–90). However, the role of BSCFB during AE remains unexplored. It is possible that either B cells or antibodies may cross this barrier more efficiently than the BBB to enter the CSF, but data from either *in vitro* studies or animal models are currently lacking (Figure 3A).

### Olfactory Route

The proximity of the OB to the nasal mucosa makes it both a vulnerable niche to insult and infection of the brain and an attractive option for delivery of therapeutics that cannot otherwise traverse the BBB (91, 92). Many viruses and bacteria co-opt this olfactory route for brain infection, and more than 40 substances have been shown to enter the brain by this means (93, 94). Viruses, such as Venezuelan equine encephalitis virus, initially infiltrate the CNS *via* olfactory axons, then induce BBB permeability throughout the brain to promote a second wave of infection, as virion particles enter the brain from the circulation *via* the damaged barrier (95). The neurotrophic Nipah virus also infects hamster olfactory sensory axons, travels into the OB, and disseminates from olfactory processing centers like the olfactory tubercle before spreading throughout the brain (96). After an initial injury to nasal mucosa, *S. aureus* infects olfactory sensory neurons and spreads to the OB *via* the olfactory axons within 6 h (97). In addition to intranasal pathogens, the olfactory route has been successfully used to deliver drugs, large proteins, and stem cells into the brain (91), largely due to rapid diffusion *via* cerebral perivascular spaces (98). Why is the olfactory route so amenable for CNS delivery? This is due to several factors: (1) diffusion across the heavily vascularized nasal mucosa that also contains lymphatic vessels, (2) direct transport by olfactory sensory axons into the OB, and (3) direct transport by the trigeminal nerve into the brainstem. Although the nasal mucosa is highly vascularized, the mean capillary density and relative permeability to hydrophilic macromolecule tracers is significantly greater in the respiratory epithelium of the nose than in olfactory sensory regions (99). Thus, sensory regions of the nasal mucosa may provide easier access to the brain due to their relatively slower clearance rates into the bloodstream (99). We have recently shown that GAS-specific Th17 lymphocytes and other T cell subtypes generated in the olfactory cavity use the sensory axon route to enter the CNS in an animal model for BGE (Figures 2 and 3B). Because T lymphocytes are primarily found in the outer nerve and glomerular layers within the OB, where incoming sensory axons form synaptic connections with projection neurons, we propose that T cells travel along the sensory axon route into the CNS (55). However, it is currently unclear whether GAS-specific T cells passively move into the brain along these sensory axons or are actively recruited into the brain by resident antigen-processing macrophages, microglia, or the olfactory ensheathing glia, the last of which are known to phagocytose bacterial debris. Olfactory ensheathing glia guide olfactory sensory axons toward their targets in the OB and thus may also serve this role for GAS-specific T cells. Once in the brain, Th17 cells likely persist due to high levels of CCR6 and LFA-1 signals, which are required for CD4^+^ T cells to remain in the CNS in the EAE model. The olfactory route is also used by immune cells to exit the CNS through the OB and drain into the deep cervical lymph nodes, in order to dampen anti-CNS immune responses in the periphery (100, 101). There are, however, no data on whether antibodies use the olfactory route to gain access to the CNS from sites of infection in the olfactory mucosa, especially for BGE associated with i.n. GAS infections.

### The BBB in Healthy and Inflamed CNS

The BBB achieves its selective permeability to proteins and immune cells by the presence of (1) TJs that prevent paracellular diffusion of small molecules and immune cells between endothelial cells, (2) very few endocytotic vesicles that restrict movement of large molecules through the transcellular pathway, and (3) transporters that shuttle select nutrients between the blood and the brain (Figure 3C) (102). The junctional transmembrane proteins claudin-3, -5, -12, and occludin are expressed at the barrier and interact in a homotypic manner to form paracellular pores that restrict diffusion between cells (Figure 3C) (102). Endocytotic caveolae in the CNS endothelium provide an essential route for receptor-mediated transcytosis (102). This process requires caveolin-1 (Cav-1), a transmembrane protein expressed at low levels within CNS blood vessels. Cav-1 levels increase during BBB breakdown following ischemic stroke, when enhanced transcytosis initiates BBB dysfunction (103). Healthy BBB vasculature also has low levels of leukocyte adhesion molecules such as VCAM-1, ICAM-1, and ICAM-2, which are upregulated on CNS vessels during neuroinflammation to promote T lymphocyte trafficking into the parenchyma (104, 105).

Just as capillaries in the CNS possess unique characteristics as compared to systemic capillaries, the BBB may be differentially porous throughout the CNS. Several studies have suggested that endothelial barriers in the brain and spinal cord are functionally different. Blood–brain and blood–spinal cord barriers differ in expression of some BBB-specific proteins and their functional permeability. Endothelial cells in the spinal cord have decreased expression of adherens junction (VE-cadherin; β-catenin) and TJ (occludin, ZO-1) proteins, as well as increased permeability to small molecular weight tracers, compared to brain endothelium (106). Endothelial cells in these two CNS regions also differ in their affinities for immune cells. Encephalitogenic T cells in the cervical spinal cord rapidly arrest on the vasculature without crawling, whereas they do not display this behavior on brain blood vessels (104). Higher expression of sphingosine 1-phosphate receptors in spinal cord ECs may facilitate preferential immune cell infiltration as compared to other CNS regions during EAE (107). Within the CNS, blood vessels in the OB and neurogenic niches also have a relatively higher permeability as compared to other CNS regions (excepting the circumventricular organs that have leaky blood vessels); blood vessels in these regions become highly permeable, in particular following viral infections (108–110), and may predispose them to infiltration of immune cells or antibodies in addition to viral particles.

Blood–brain barrier function can be impaired by several factors including inflammatory cytokines, hormones (e.g., epinephrine), and substances of abuse (e.g., alcohol, cocaine, and methamphetamine) (31, 102, 111). Several inflammatory cytokines including IL-1β, TNF-α, CCL-2, and IL-17A, which are present in the CNS or blood during neuroinflammation, affect the stability of the BBB by either degrading TJ proteins, modifying their phosphorylation states, or affecting the turnover rate (Figure 3C). IL-1β indirectly destabilizes the BBB by inducing expression of matrix metalloproteinase-9 and VEGF, two factors that promote degradation of Claudin-5, Occludin, and ZO-1 (112, 113). TNF-α also induces formation of gaps between cell junctions by upregulating transcription of NF-κB and myosin light chain kinase, a factor known to internalize TJ proteins *via* caveolae (114, 115). The chemokine CCL-2 produced by macrophages also enhances BBB permeability by inducing phosphorylation of occludin and claudin-5 and promoting their endocytosis *via* caveolae (116). IL-17 and IL-22 produced by Th17 cells in EAE/MS are also known to disrupt endothelial cell TJs (117). Although these cytokines have been shown to disrupt barrier function during neuroinflammation, it is unclear whether they are present in AE. Recent cytokine profiling of CSF from patients with viral and autoimmune encephalitis revealed that several Th1 cytokines, such TNF-α, IFN-γ, CXCL9, and CXCL10, are elevated during viral encephalitis, but not in AE samples. In contrast, IL-6, a cytokine essential for development of Th17 cells, is elevated in all CNS autoimmune disorders (11). The predominant mechanism for BBB disruption in our animal model for *S. pyogenes*-induced BGE appears to be disruption of endothelial TJs *via* formation of gaps and protrusions that enhance BBB permeability and promote entry of autoantibodies into the CNS (55). Although we cannot exclude other factors such as cytokines released by activated microglia/macrophages that are present in the CNS after multiple i.n. GAS infections (55), or the ability of GAS antibodies to break down endothelial cell junctions, the high degree of correlation between the presence of bacterial-specific Th17 cells and BBB leakage suggests that IL-17 likely contributes to BBB dysfunction in a similar fashion as in EAE/MS (55, 115). *S. pyogenes*-specific Th17 cells are present in the tonsils of human patients (55); however, it remains to be shown whether they are important for human disease pathogenesis.

B cells can arrest along endothelial cell walls using the same adhesion mechanisms as T cells (VLA-4- and ICAM-1-mediated arrest and adhesion). In addition, B cells can infiltrate into the CNS, by extravasation through disrupted TJs, reminiscent of T cell entry across the BBB (118–120). However, the number of B cells that has been reported to present in the CNS following histological analyses of patients with GBS, BBE, MFS, or any of the animal models of AE discussed above is indeed extremely small compared to the number of T cells (77). B cells can infiltrate the inflamed CNS in MS and SLE patients and form ectopic lymphoid structures, or cellular aggregates outside germinal centers, which reinforce the immune response locally (90, 121–123). In MS, B cells within cellular aggregates present antigens to T cells, contribute to epitope spreading, and produce antibodies that are detectable as oligoclonal bands in the CSF collected from patients (123). However, studies using mouse models of SLE have produced confounding results as to whether B cells that are resident within the CNS secrete autoantibodies (89, 124). In addition, there is scant evidence for B cell infiltration during AE. It is possible that these diseases are mediated by locally infiltrating B or plasma cells (125), but firm evidence for CNS-resident B cell populations is still lacking.

Endothelial cells are also highly vulnerable to endotoxins secreted by infectious agents. Both antigenic surface proteins on Gram-positive bacteria and lipopolysaccharide from Gram-negative bacteria change endothelial barrier properties (126). Environmental toxins and food additives can also increase BBB permeability, as evidenced by serum protein leakage after bis(tributyltin) oxide exposure (127). Finally, the gut microbiome may also affect the function of the barrier (128). Given the presence of many anti-neuronal autoantibodies in circulation, transient BBB permeability from any of these sources, or reactivated F_c_ receptor-mediated transcytosis through blood vessels (Figure 3D), would provide opportunistic access to brain targets. Pathological antibodies may also enhance BBB permeability, promote production of inflammatory cytokines, and stimulate adhesion and migration of immune cells across the CNS. For example, anti-NR2 glutamate receptor antibodies (anti-NR2) derived from patients with SLE promote expression of both VCAM-1 and ICAM-1 and increase the production of IL-6 in brain endothelium that promotes BBB inflammation and changes to its permeability (129). Antibacterial antibodies can also change endothelial barrier permeability; however it is unknown whether this mechanism mediates antibody entry into the CNS during AE. Furthermore, rare cases of GBS with CNS lesions provide insight into how the same bacterial infection may cause central as opposed to peripheral autoimmunity (77, 78). CNS lesions in the brain (77, 78) and spinal cord (77), as well as CSF pleiocytosis or excess protein (130), together with the presence of immune cell infiltration in both CNS regions (77) indicate immune involvement in GBS infections of the CNS. Active forebrain lesions in the patient described by Okumura are compelling evidence for a BBB breach (78). Cytokine-producing CD4^+^ and CD8^+^ immune cells in the CNS were mostly confined to dense clusters in the meninges and perivascular cuffs, but a population of CD4^+^ and CD8^+^ T cells were found in the brainstem with a very few B cells intermixed in perivascular immune cell infiltrates (77). Therefore, brain lesions or T cell infiltration into perivascular spaces may be important steps in breaching the BBB and thereby providing antibody access to the brainstem, for certain GBS subtypes.

## Treatment of AE Syndromes

The treatments currently employed for BGE focus on immunotherapy to weaken the autoimmune response. Screening for cancer is essential in ruling out a neoplastic syndrome; if a tumor is present, surgical interventions are effective in alleviating symptoms for 75% of NMDAR encephalitis cases. In cases without a tumor, first-line treatment with immunotherapy using high doses of corticosteroids, plasma exchange, and/or IVIg improves patient outcomes in two-thirds of cases. Cases that do not respond to primary immunotherapy receive treatment with Rituximab (a monoclonal antibody that neutralizes B cells, thereby halting antibody production) or cyclophosphamide (a potent immunosuppressant) (72). EEG monitoring and antiepileptic treatments are frequently necessary in AE; seizures are common with encephalitides of autoimmune and infectious origin (13) and may trigger BBB leakage due to release of the potent inflammatory cytokines IL-1 and TNF-α from dying neurons. Controlling further seizures is paramount to stave off permanent damage, especially for pediatric patients.

Encephalitis with an infectious trigger such as *S. pyogenes* warrants primary treatment with antibiotics to eliminate the latent infection (131). First-line treatment for SC or PANDAS, both of which are associated with streptococcal infections, centers on eliminating autoantibodies from circulation by means of plasma exchange or plasma apheresis, coupled with corticosteroid treatment. Plasma exchange shows promise as a treatment option, but IVIg has only a minor effect (29, 30). A recent double-blind clinical study of IVIg for 35 PANDAS patients found no improvement compared to placebo after two rounds of IVIg (132). Plasmapheresis is a beneficial, if invasive, treatment option, with an average improvement of 65%, 6 months after treatment (30). Indeed, many immunotherapies recommended for AE require several weeks or months to show effectiveness.

Outcomes are generally good in younger patients, including children. Young adult and pediatric autoimmune encephalitis patients have a recovery rate of up to 80%, although improvements continue slowly for up to 2 years. In patients who recovered from NMDAR encephalitis, the relapse rate is around 25%, so yearly screening for tumors is recommended after recovery (133). After surgical excision of tumors, if warranted, following up by treatment with immunotherapy leads to generally good outcomes for NMDAR encephalitis. PANDAS patients typically also recover after treatment and learn to manage their exposure to *S. pyogenes*. Some patients continue prophylactic antibiotics to minimize such exposure, sometimes for several years after their most recent relapse.

For any type of AE, studying BBB function during both disease initiation and periods of remission would greatly clarify the role of the barrier over the course of disease and help inform treatment options. MRI with gadolinium enhancement is a standard tool for MS diagnosis and disease monitoring (102, 134) and is a sensitive diagnostic tool for GBS. Based on our studies on the animal model for *S. pyogenes*-induced BGE, we argue that MRI with gadolinium enhancement may reveal latent BBB damage in AE patients or foci of BBB damage (135–137). Our working hypothesis that barrier breakdown is a crucial step during AE pathogenesis would, therefore, be strengthened by evidence from patient MRI with gadolinium enhancement or careful analysis of BBB integrity using intravenous fluorescently labeled low- or high-molecular-weight tracers in animal models for other types of AE (55).

Future treatment avenues may rely on modulating BBB permeability using biological or chemical therapeutics. One exciting option is reactivation of signaling pathways that normally function to form the BBB during development, in order to repair the dysfunctional barrier during disease. The barrier properties of CNS vessels develop prenatally in most CNS regions for both mice and humans with the exception of the retina, which vascularizes postnatally in mice. Wnt/β-catenin signaling promotes both CNS angiogenesis and BBB formation by production of Wnts from neural progenitors (138–142). Wnts also induce expression of some EC-specific proteins, including the glucose transporter Glut-1 and several TJ components (138–142). Following angiogenesis, Hedgehog signaling is required for acquisition of barrier properties in CNS ECs, including expression of TJ proteins occludin and claudin-5 (142, 143). Mature blood vessels continue to respond to Wnt and Shh cues, indicating that these pathways are active in maintaining EC barrier properties (139, 142, 143). Recently, Wnt signaling was reported to be upregulated in both EAE and human MS lesions, correlating with increased neuronal Wnt3 expression and TJ breakdown. Reactivation of Wnt signaling in EAE may serve to repair the damaged BBB in inflammation. Inhibition of Wnt signaling hastened disease progression and resulted in increased numbers of CD4^+^ T cells in the CNS (105). Chemical modulators of Wnt signaling have been validated *in vitro* and in rodent models of neuroinflammation (144, 145). Translation of such therapies to clinical use could repair the damaged barrier, translating to improved clinical outcomes in AE patients by limiting influx of blood-borne immune cells, cytokines, and/or antibodies.

## Conclusion

Autoimmunity in the CNS remains confoundingly complex, in both etiology and treatment. While triggers for AE are defined in some cases, frequently, no clear infectious or cancerous cause can be found. BBB integrity clearly plays a role in disease development, and more research on differential barrier permeability over the course of disease would resolve many questions about autoantibody entry and pathogenesis. Animal models for these diseases have the potential to uncover new routes of antibody entry that are not apparent from patient imaging studies. Indeed, infectious triggers themselves may have the inherent ability to disrupt the BBB to allow access to brain antigens. By approaching AE from several angles, a more complete picture of pathogenesis, contributions to symptom exacerbations, and recommended treatment options will emerge.

## Author Contributions

MP, DA, and TC conceived of the topic and wrote the manuscript, and MP prepared figures and figure legends.

## Conflict of Interest Statement

The authors declare that the research was conducted in the absence of any commercial or financial relationships that could be construed as a potential conflict of interest.
